# A review of physiological and cellular mechanisms underlying fibrotic postoperative adhesion

**DOI:** 10.7150/ijbs.54403

**Published:** 2021-01-01

**Authors:** Qiongyuan Hu, Xuefeng Xia, Xing Kang, Peng Song, Zhijian Liu, Meng Wang, Xiaofeng Lu, Wenxian Guan, Song Liu

**Affiliations:** Department of Gastrointestinal Surgery, Nanjing Drum Tower Hospital, the Affiliated Hospital of Nanjing University Medical School.

**Keywords:** adhesion formation, mesothelial cells, fibrosis, immune response

## Abstract

Postoperative adhesions (PA) are fibrotic tissues that are the most common driver of long-term morbidity after abdominal and pelvic surgery. The optimal drug or material to prevent adhesion formation has not yet been discovered. Comprehensive understanding of cellular and molecular mechanisms of adhesion process stimulates the design of future anti-adhesive strategies. Recently, disruption of peritoneal mesothelial cells were suggested as the 'motor' of PA formation, followed by a cascade of events (coagulation, inflammation, fibrinolysis) and influx of various immune cells, ultimately leading to a fibrous exudate. We showed that a variety of immune cells were recruited into adhesive peritoneal tissues in patients with small bowel obstruction caused by PA. The interactions among various types of immune cells contribute to PA development following peritoneal trauma. Our review focuses on the specific role of different immune cells in cellular and humoral mechanisms underpinning adhesion development.

## Introduction

Most patients develop postoperative adhesions (PA) following abdominal surgery or pelvic surgery, and PA has great influence on socioeconomic cost and living quality of millions of patients worldwide. Severe PA can cause serious complication, including small bowel obstruction (SBO), chronic abdominal pain, and even female infertility 1. Adhesiolysis at repeated surgery leads to increased mortality, infectious complication, longer hospital stay, and increased clinical burden 2. The prevention of PA and awareness of adhesion-related morbidity during adhesionlysis deserve priority in clinical and experimental practice.

PA formation is still a clinical challenge today. Current strategies on prevention of adhesion are based on the usage of physical barrier or through careful surgical procedures, but there is no clinical evidence that these strategies improve the adhesion-associated complications 3. The reason is that the pathogenesis is not well understood. Therefore, a comprehensive understanding of cellular and molecular mechanisms of PA process allows the design of new anti-adhesive strategies. Therefore, our review focuses on the role of various immune cells in the adhesion of the abdominal cavity.

## The peritoneum

**Figure 1:**
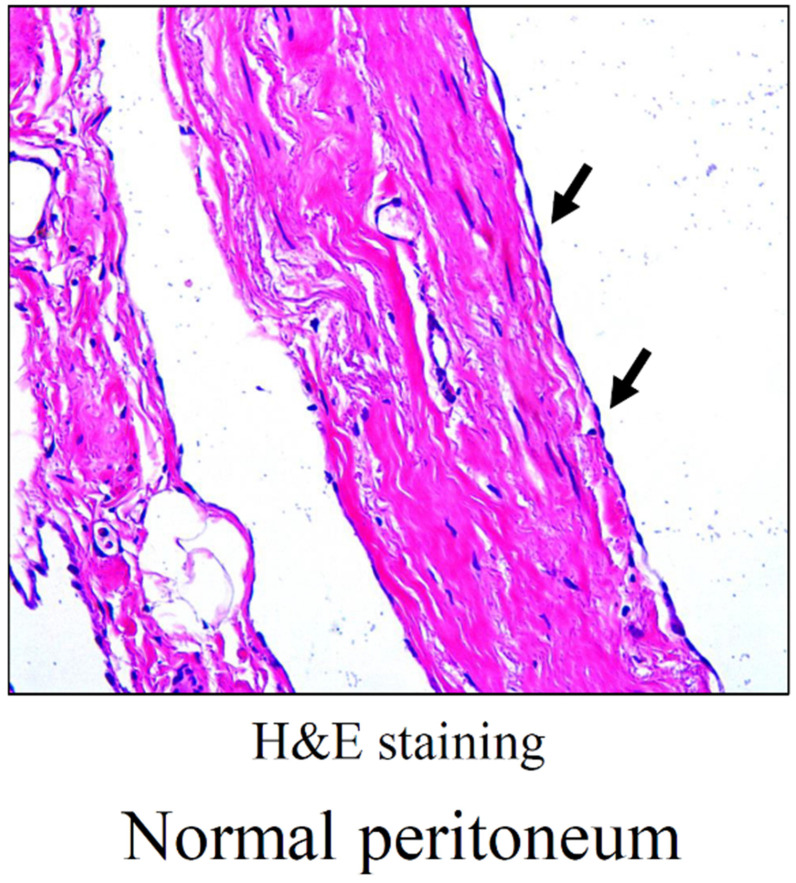
Morphology of normal peritoneum. Representative image of HE staining for normal peritoneum. Arrows point to mesothelial monolayer.

Compared with pericardium and pleura, peritoneum is the most extensive serous membrane in body. Peritoneum is a serious membrane covered by a monolayer of flat, microvilli-rich mesothelial cells (**Figure 1**). As mesothelial cells are poorly interconnected via very loose intercellular bridges, the peritoneum is highly susceptible to trauma. Recently, normal peritoneum was dissociated for single-cell transcriptomes, and they firstly categorized peritoneal cell types of normal peritoneum and assigned peritoneal cells to seven distinct cell types, including mesothelial cell (61.5%), fibroblast (16.8%), endothelial cell (10.4%), myofibroblast (6.1%), mononuclear phagocyte (3.8%), B lymphocyte (0.9%), T lymphocyte (0.5%) 4.

It is reported that the frictionless surface of the mesothelium, the epithelial monolayer, lines the peritoneal cavity and visceral organs, exert a protective role against PA 5. Therefore, adhesion formation requires damage of mesothelium integrity, and exposure of basement membrane is crucial for the fibrin attachments between denuded surfaces. Recent study demonstrated that genes associated with extracellular matrix (ECM) formation were down-regulated within 24 hours after peritoneal injury, and they suggested that down regulation of ECM proteins and collagen at early stage of adhesion formation enables the mesothelium to move into the peritoneal cavity 6.

## Physiological mechanism of PAF

### Apoptosis and proliferation

**Figure 2:**
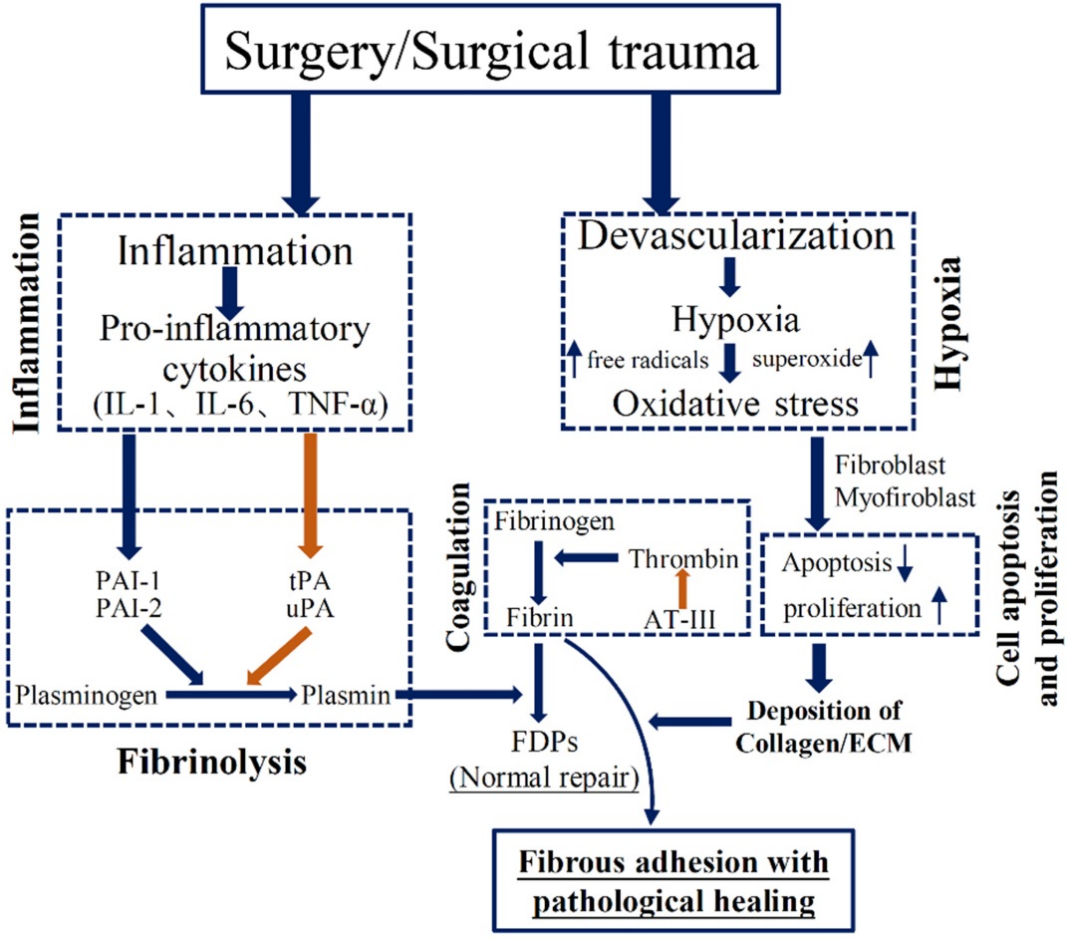
Brief schematic illustration of the pathogenesis of adhesion formation. Interaction of inflammation, oxidative stress, fibrinolysis, coagulation, and cell apoptosis and proliferation in adhesion development.

The process of apoptosis and proliferation has been indicated to play a vital role in PA development (**Figure 2**). Tissue hypoxia could disrupt the balance of cellular differentiation, proliferation, and death, which could lead to impaired repair process in peritoneal wound. Interestingly, hypoxia was demonstrated to induce apoptosis in normal peritoneal fibroblasts, but decrease apoptosis index in adhesion fibroblasts 7. In particular, when adhesion fibroblast was exposure to hypoxia, it had markedly higher proliferation. Mitochondrial signaling pathways are involved in the induction of apoptosis during tissue hypoxia, and some specific genes, including p53, Bcl-2 family, and caspases, were suggested to stimulate the apoptotic signals 7. Recent study used a model of early adhesion formation to show the induction of genes responsible for proliferation and inhibition of genes for apoptosis in the peritoneal mesothelium after trauma 6.

### Oxidative stress

Tissue hypoxia could contribute to the increased oxidative stress, with enhanced generation of nitrogen and oxygen free radicals, which leads to DNA damage and increased production of oxidized protein 8. During the first 5 minutes after hypoxia, free radicals was significantly produced via an increased formation of reactive oxygen species (ROS) 9. Free radicals should be controlled to prevent tissue injury and are regulated through the antioxidant enzymes. The imbalance between the production of free radicals and the defense mechanisms of antioxidant enzymes leads to the oxidative stress, which was suggested as a predisposition to the adhesion phenotype (**Figure 2**) 8. These free radicals were demonstrated to promote the expression of many factors, including transforming growth factor *β*, IL-6, type I collagen, and vascular endothelial grow faction 10. Additionally, tissue hypoxia acutely induces the production of superoxide. Fibroblasts exposed to the superoxide could produce pro-fibrogenic factors, such as TGF-β and collagen type I 11. From previous studies, oxidative stress damage was also implicated in the activation of matrix metalloproteinases (MMPs). MMPs are proteolytic enzymes that are responsible for the remodeling of ECM. Human peritoneal cells (mesothelial cells, fibroblasts) are suggested to synthesize MMPs in order to degrade excessive ECM, and the serum level of MMPs is a potential biomarker of surgical adhesion 12. ROS could induce the activation of transcription factors which can activate MMPs activity. A clinical finding suggested a positive correlation with the relative levels of MMPs and the degree of oxidative stress 13. Using an *in vivo* mouse model of PA, the production of ROS was accompanied by an increased activity of MMPs. Many antioxidants that scavenge free radicals are suggested to prevent the development of PA phenotype, including N-acetyl-cysteine 14, lycopene 15, and emodin 16. Detailed biological mechanisms may help to enlighten our understanding of the relationship between oxidative stress and adhesion process. Furthermore, ensuing meticulous hemostasis and avoiding air drying of exposed tissues could diminish the adhesion development 17.

### Inflammatory responses

The severity of acute inflammation is associated with enhanced adhesion formation (**Figure 2**). Tsai et al. 6 recently performed RNA sequencing in isolated surface mesothelium using an *in vivo* mouse model of adhesion. They showed that expressions of genes set associated with inflammation response encoding cytokines, chemotactic factors, and nuclear factor κB signaling components, are early regulated following the induction of adhesion. Interestingly, genes involved in the ECM deposition were down-regulated within 24 hours after injury, including TGF signaling, fibronectin, and collagens 3. An increased production of inflammatory mediators in the early stage plays an important role in regulating ECM formation during PA 18. Increased tumor necrosis factor-alpha (TNF-α) concentration is involved in the repair progress during surgical adhesion. The mRNA expression of TNF-α is increased by 58% in adhesion fibroblasts as compared to normal fibroblasts 19. Like TNF-α, interleukin-6 (IL-6) is pro-inflammatory cytokine, leading to systemic inflammatory reaction. Both TNF-α and IL-6 were suggested to regulate the formation of coagulation cascade and fibrin formation 19. Uyama el al. 18 recently suggested that treatment with IL-6 receptor antibody alleviated surgical adhesion formation. Other inflammatory mediators, such as IL-17 and IFN-γ, also served as potential therapeutic target molecules for prevention of surgical adhesion 20-22. Collectively, the extent of injury determines the degree of the inflammatory response; the degree of inflammatory reaction in turn determines the severity of adhesion formation.

### Coagulation and Fibrinolysis

Extensive tissue injury, hypoxia, and the inadequate fibrinolytic activity of the peritoneum contribute to an imbalance between pro-coagulatory and fibrinolytic reaction, inducing the formation of fibrin clots (**Figure 2**). Once hypoxia damages the peritoneum, the coagulation cascade is altered and, eventually induced the formation of fibrinous matrix and fibrin bands. Thrombin is the final enzyme of coagulation cascade and transfers fibrin into fibrin monomers. In normal condition, the fibrin bands could be degraded into smaller molecules (FDPs) by fibrinolysis that is regulated by enzyme plasmin. Plasmin is originated from urokinase-like plasminogen activator and tissue-type plasminogen activator (tPA). Meanwhile, to keep the balance, tPA could be regulated by plasminogen activator inhibitor-1 (PAI-1) 23. When the severe peritoneal injury is caused by abdominal surgery, the imbalance between tPA and PAI-1 could lead to increased fibrin exudate and persistent fibrinous mass 23. Another vital player, antithrombin III poses anticoagulation effect by decreasing thrombin activity 5. Thus the likelihood that fibrinous collections at surgical sites would undergo fibrinolysis is markedly reduced, and subsequently fibroblasts migrate into the persistent fibrinous connective mass, leading to production of ECM and can induce the adhesion formation 24. Increased inflammatory response (IL-1, IL-6, and TNF-α) could down-regulate tPA activity, thereby decreasing the tPA/PAI ratio and fibrinolytic activity, and leading to the adhesion formation 25.

## Cellular mechanisms of PA

**Figure 3:**
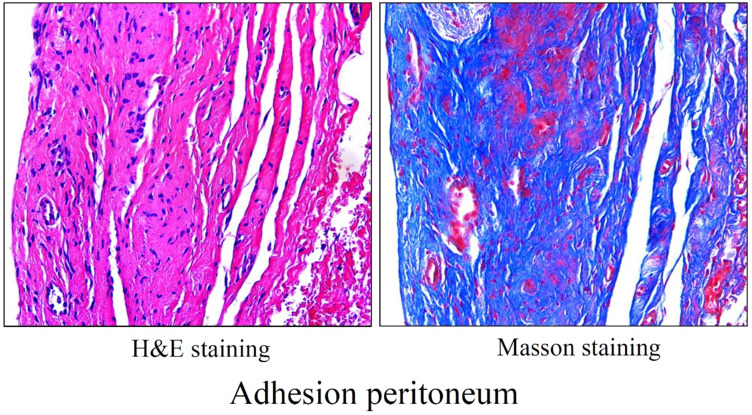
Morphology of adhesion peritoneum. H&E staining (left) and Masson staining (right) of representative human adhesion tissue.

**Figure 4:**
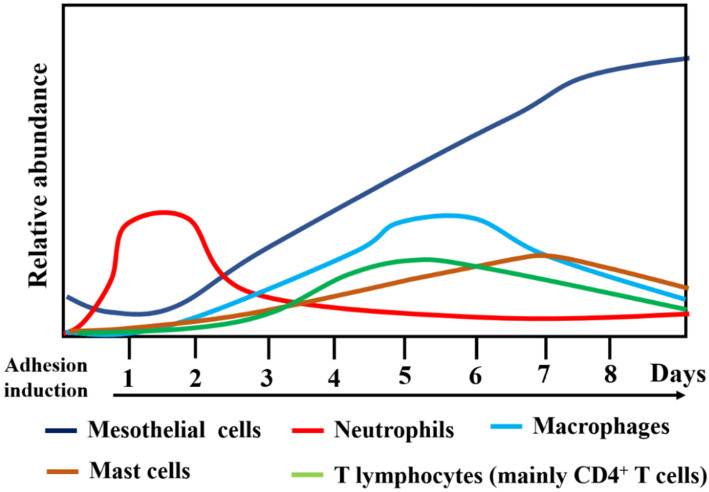
Changes in the relative abundance and type of cells types during surgical adhesion development.

The human adhesive tissues were markedly thickened, and filled with various cells by H&E staining, and Masson staining showed increased collagen deposition in the adhesive peritoneum (**Figure 3**). In addition to mesothelial and endothelial cells, and fibroblasts, a variety of inflammatory cells corresponding to neutrophils, macrophages, lymphocytes, mast cells were also present. The interactions among various types of immune cells could contribute to PA following peritoneal trauma, and these various cell types play different roles at different stages (**Figure 4**). Therefore, it is important to understand the role of every cell type in adhesion phenotype.

### Mesothelial cells (MCs)

Mesothelial cells play a vital role in tissue repair and inflammatory process through secretion of inflammatory cytokines, grow factors, and ECM factors. Recently, Fischer et al. 26 suggested that profuse membrane bridges between mesothelial surfaces initiate adhesions, implicating that pathological changes of MCs acts as the main component of early adhesion cascade.

Previous studies suggested that in response to peritoneal injury, MCs can transdifferentiate into a subset of myofibroblasts via mesothelial-to-mesenchymal transition (MMT) 27,28. Under pathological circumstances, activated MCs showed loss of epithelial function and acquired myofiroblastic phenotype to migrate to the submesothelial stroma, where they released collagen and ECM components. Sandoval et al. 29 revealed that mesenchymal transition of MCs participated in the development of adhesion formation. The transition of MCs into myofibroblasts has also been described in pleural tissue 30. Consistent with this, Uyama et al. 18 demonstrated that adhesion-associated myofibroblasts are manly originated from MCs, generating the adhesion band. Angiogenesis is another important morphological character of fibrous band, and high secretion of VEGF is an important factor in leading to vascularization during adhesion formation 31. Previous evidence suggested that the MCs could secret a large amount of VEGF during MMT process. Many animal experiments showed that treatments against MMT prevented the development of peritoneal fibrosis and angiogenesis, protecting peritoneal structure and function 32,33. Strippoli et al. 34 showed that caveolin1 and YAP drive mechanically induced mesothelial to mesenchymal transition during adhesion development. Therefore, modulating MMT procedure could be a potential target in reduction of PA formation.

Acute inflammatory response requires fast migration of inflammatory cells to the injured site through production of chemokines and interaction with integrins or cell adhesive molecules 19. Different adhesion molecules secreted by MCs may recruit the specific type of cells on the surface of MCs. Wang et al. 35 showed that the recruitment of Th1 cell on human MCs monolayers was regulated by α6β1 integrin while that of Th2 cell was regulated by anti-α4 integrin. Additionally, production of arginase by MCs could regulate the activity of CD4^+^ T lymphocytes *in vitro* 36. MCs also express MHC II molecules and can therefore modulate the antigen presentation 37. MCs also participate in the inflammatory and tissue repair process through secretion of a wide range of cytokines, growth factors, and ECM molecules 10. 0.1%-0.5% of MCs undergoes mitosis at normal condition; however, increased rate (30%-60%) in mitosis was showed in injured peritoneum, indicating increased production of cytokines and growth factor by MCs 38. Activated MCs could form MCs cell islands through rapid proliferation. They continue proliferating until the MC islands connect with each other to complete the mesothelial repair, which usually takes 5-7 days 39,40.

Much is known about the later stage of PA formation involving fibrinolysis and fibrin deposition, but the molecular and mechanical details of initial stages are largely unknown. Tsai et al. 6 recently demonstrated that activated MCs serve as a motor role during PA using multiple lineage-tracing approaches, and preventive therapeutic method targeting MCs led to reduction of adhesion formation in a mouse model. Unexpectedly, TGF-β was down-regulated in the MCs, indicating the later role of TGF-β in adhesion or released by other types of cells. They suggested that the decreased of collagens and ECM proteins promoted the mesothelium to detach from the base membrane and reach into peritoneal space. Mechanically, MSLN^+^ MCs is the important subpopulation of MCs involved in the development of adhesion. Foster et al. 41 recently found that JUN expression was strongly induced and correlated with prominent MSLN expression, and inhibition of JUN significantly minimizes adhesion formation.

### Neutrophils

Inflammation plays an important role in PA formation, and initiation of an increased inflammatory response requires recruitment of inflammatory cells into the surface of MCs. Vural et al. 42 suggested that neutrophils were fast recruited into the injured site from the circulation. The first cells that migrated into the injured area were polymorphonuclear leucocytes, which can persist for 2 days 38. Using *in vivo* experimental adhesion model, Ly6G^+^ neutrophils were recruited into the injured serosa, peaking in numbers following cecal cauterization 18. However, macrophages, T cells, and B cells were not showed to accumulate in the injured site. Recently, Uyama et al. 18 demonstrated that neutrophil-ablated mice by administration of anti-Ly6G monoclonal antibody showed reduced adhesion phenotype following adhesion induction, hereby confirming the crucial role of neutrophils in the development of adhesion phenotype.

Selective reduction of numbers in neutrophils could possibly lead to systemic effect on the number of circulating neutrophil, contributing to an immune compromised patient during peri-operative period 43. Therefore, it is important to understand the potential mechanisms by which the accumulated neutrophils induce adhesion phenotype. Accumulated neutrophils were suggested to enhance production of ROS (mainly superoxide radicals), and prevention of ROS generation by the neutrophils at the injured site helped to avert adhesion formation 44. A recent study observed that TGF-β^+^Ly6G**^+^** neutrophils were showed in the injured serosa and adjunct submucosa at the early stage of adhesion induction, indicating that recruited neutrophils at the injured site could produce TGF-β 18. TGF-β is a well-established master inducer of fibrosis and adhesion formation 45. Therefore, neutrophils are very important in contributing to the transition from pro-inflammatory response to pro-fibrotic condition. Additionally, a process named 'NETosis' (a process that neutrophils can exclude neutrophil extracellular traps) for neutrophils has been recently found to contribute to the adhesion formation. Tsai et al. 46 analyzed the sections of adhesive tissues by microscopy, and demonstrated that neutrophils in adhesion sites differed in morphology (more elongated) from traditional neutrophils (small, circular cells), indicating that neutrophils were not only simply migrating from circulation into the injured sites, but also possibly experiencing some changes. They further observed that circulating neutrophils could undergo NETosis after recruitment by mesothelium. However, disruption of NETosis with DNase is not sufficient to prevent adhesion formation.

### Macrophages

Previous evidence suggests that peritoneal macrophages are crucial in the reconstitution of peritoneum after trauma. Macrophages are the most prevalent cell type within the injured peritoneum; previous data showed that macrophages were present on the day 1 after adhesion induction 47. On the day 5 or 6, most of the injured site was covered by newly mesothelial cells, and the amount of macrophages significantly decreased 48. Macrophages could produce plasminogen activator inhibitors and tissue plasminogen activators that modulate fibrinolysis and inflammatory response 49. A recent study demonstrated that although activated MCs induced the recruitment of monocytes through monocyte chemoattractant protein (MCP), the number of accumulated macrophages was markedly decreased throughout the adhesive time course 46. They showed that neutrophils and macrophages play opposite role in the development of adhesion formation. Macrophages play a protective role in the pathogenesis of adhesion though executing programmed cell removal, phagocytosing apoptotic neutrophils. Additionally, recruitment of monocytes with MCP and thioglycolate significantly contributed to a moderate induction of adhesion burden. Rajab et al. 50 also suggested that enhanced peritoneal macrophages by protease peptone increased the activity of plasminogen and inhibited the development of adhesion formation. Additionally, macrophage depletion and an irritation/injury of the peritoneum resulted in peritoneal adhesion formation in a mouse model 49. A previous study suggested that prior depletion of macrophages could induce a significant neutrophil influx into the tissue in response to lipopolysaccharide 51.

The above findings suggest an important role of macrophages in adhesion phenotype, but their differentiation is still unknown. Macrophage function depends mainly on differentiation status, and its differentiation could affect wound healing in several organs 52. M1 macrophages play an important part in the degradation of the ECM during inflammatory response 53. However, M2a subtype participates in wound healing and tissue remodeling by producing ECM 54,55. Another subpopulation, the M2b macrophages are demonstrated to play an important role in modulating immune and inflammatory response and thereby decreasing tissue injury 56. The third subpopulation of M2, the M2c macrophages participate in the immune suppression and degradation of ECM. Hong et al. 57 suggested that macrophage polarization has tremendous effect during adhesion. They showed that there was an inverse correlation between M2 marker expression and adhesion formation. Mechanically, macrophage-specific peroxisome proliferator-activated receptor γ reduces surgical adhesion formation by modulating arginase activity and macrophage polarization. Base on the previous findings, the role of macrophage differentiation on fibrosis and adhesion formation is still controversial. Therefore, understanding of the functions and mechanisms of the various macrophage subpopulations can help to open up a new strategy for the prevention of surgical adhesion formation.

### Mast cells

Mast cells in peritoneal cavity are tissue-type mast cells. Those present in peritoneal fluid can be derived from the intestinal wall. Mast cells are the important effector cells of inflammation and play a vital role in tissue reparative reactions and immune response 58. They react to a variety of stimuli and are degranulated when an inflammatory process occurs. Moreover, mast cells have been suggested to contribute to the healing process and tissue remodeling 59. Previous studies suggested that the number of mast cells were increased in the late stage of healing process. Adam et al. investigated that mast cells expanded during adhesion induction, and their percentage showed the highest after 168 hours after laparotomy 48, which indicated that mast cells influenced the adhesion phenotype and possibly contributed to adhesion remodeling. Furthermore, reduction of mast cells has been suggested to prevent adhesion formation 58. Mast cells are shown to be responsible for the release of VEGF following adhesion induction 60. Additionally, mast cells could participate in fibrosis by production of histamine, tryptase, TGF-beta, collagen or TNF-alpha 48,61,62.

### T lymphocytes

T lymphocytes are suggested to regulate inflammatory and chemotactic response, which participate in fibrinogenic tissue disorders 63,64. Infiltration of T-lymphocytes (CD45) were found in the specimens of patients that suffered PA 65. Chung et al. 66 firstly showed T cells and T cell-derived factors played an important role in the pathological process of adhesion formation. Mechanically, this adhesive process was primarily regulated by T helper type 1 (Th1) cells and was associated with the production of IL-17 and CXC chemokines macrophage inflammatory protein and neutrophil chemoattractant released by T cells. Their further study investigated that programmed death-1 inhibitory pathway was responsible for the CD4^+^ infiltration during adhesion formation and cytokine production 67. Because Th1 cells were central to adhesion phenotype, Tzianabos et al. 68 identified the key regulators of Th1 cells activation and differentiation. Tim-3, the surface marker of Th1 cells, is crucial to recruit Th1 cells, by IL-16, with subsequent elaboration of IFN-γ. Additionally, the transcription factor T-bet is the vital regulator of Th1 cell differentiation. Tzianabos et al. 68 also demonstrated that T-bet-deficient mice were resistant to develop severe adhesion following cecal cauterization. From the above findings, Th1 cells and their released cytokines play an important role in the pathogenesis of PA formation.

CD4^+^ T cells consist of conventional NK1.1^-^CD4^+^ T cells and natural killer T (NKT) cells expressing αβ T cell receptor 69. NKT cells were recruited in the injured site to induce inflammatory cascade and subsequent tissue injury 64. Kosaka et al. 22 investigated that NKT cells, rather than Th1 cells, were shown to be essential for intestinal adhesion formation. They demonstrated that NKT cell-deficient mice developed adhesion poorly, whereas reconstitution with NKT cells in wild-type mice induced severe adhesion phenotype. They also suggested that INF-γ derived from NKT cells was indispensable for adhesion phenotype, providing a new therapeutic target molecule. Their further results suggested INF-γ caused adhesion formation through the reciprocal balance between plasminogen activator inhibitor and tissue plasminogen activator 21, the key mediators in fibrinolytic activity 70. It is believed that CD4^+^ T cells are really important to promote and maintain surgical adhesion, but which subpopulation that works needs to be clarified.

As described above, those cell types are all contributors to the development of adhesion formation, but other cell types, such as eosinophils 48, and B-lymphocytes 71 may also contribute to adhesion. These immune cells may interface and/or signal with the mesothelium before the irrevocable outward expansion of the mesothelial cell progeny deposits in adhesive fibrosis. Further studies will need to specifically label these cells chemically and genetically, and then traced these cells following the induction of adhesion to clarify their precise function and contributions to adhesion phenotype.

## Adhesion Prevention and PA models

Massive efforts, including mechanical barrier or administration of pharmacological agents, have been applied to prevent adhesion formation. They provide a physical barrier between injured tissues preventing their apposition; or they inhibit either the inflammatory cascade or the fibrin-forming process during surgical trauma. Several factors have been the major deterrents in the application of mechanical barriers into clinical practice, including difficulties in preparation and application, the need for absolute hemostasis, insufficient pliability, intricate product fixation techniques, and incompatibility with laparoscopic surgical procedure 72. Even if several barrier materials have been clinically used, PA is still responsible for relevant complications following abdominal surgery. Therefore, it is important to clearly understand the potential biological function that how these mechanical barriers interfere with intra-abdominal wound healing. Additionally, most pharmacological agents on PA prevention were performed using animal models. So far, there has been no available therapeutic drug in clinical practice. The future of adhesion prevention strategy possibly has the most promise in a device that combines a barrier with targeted biological efficacy.

A reliable PA model is important to investigate biological mechanisms and anti-PA strategies. There are two classic PA models: cecum-sidewall model (CSM) and ischemic buttons model (IBM). The CSM is to rub the cecum and the adjacent abdominal wall with a scalpel, brush, or dry gauze to punctate bleeding, followed by closure 40,73. CSM is sufficient to induce damage, but the size of wound and the degree of friction are difficult to control, contributing to the instability of PA. For IBM, ischemic buttons are placed on the peritoneal wall by clamping a small (~5 mm diameter) piece of peritoneum with a hemostat and ligating the base with a 4-0 silk suture 6,73. The resulting ischemia will cause inflammation, leading to adhesions. However, Wolfgang et al. suggested that adhesion are easily prevented by barrier for CSM, which causes a risk of overstating their anti-PA ability 73.

## Conclusion

Complications of PA formation are frequent, have a large negative effect on patients' health, and increase workload in clinical practice 74. Recent therapeutic strategies focus on the usage of physical barrier during PA formation in clinic, but the optimal material to reduce adhesion has not yet been discovered. Therefore, more effective prevention techniques are most likely to evolve from a deep understanding of the molecular and cellular mechanisms underlying PA formation. Our review provides a throughout understanding of every type of immune cells in the development of surgical adhesion.
